# Joint association of serum sodium and frailty with mild cognitive impairment among hospitalized older adults with chronic diseases: a cross-sectional study

**DOI:** 10.3389/fnut.2024.1467751

**Published:** 2024-10-21

**Authors:** Zhaozhao Hui, Lina Wang, Jing Deng, Feng Liu, Liping Cheng, Yajing Li, Yuxin Tian, Le Ma, Xiaohong Liu

**Affiliations:** ^1^School of Public Health, Xi’an Jiaotong University Health Science Center, Xi’an, China; ^2^The First Affiliated Hospital of Xi’an Jiaotong University, Xi’an, China; ^3^Tongchuan People’s Hospital, Tongchuan, China

**Keywords:** mild cognitive impairment, serum sodium, frailty, older adults, chronic disease

## Abstract

**Background:**

To examine the associations of serum sodium and frailty with the risk of mild cognitive impairment (MCI) among hospitalized older adults with chronic diseases.

**Methods:**

A cross-sectional study was conducted in 403 hospitalized older adults with chronic diseases. Serum sodium concentration was assessed by the ion-selective electrode method, frailty status was evaluated by the FRAIL scale, and MCI was determined by the Montreal Cognitive Assessment (MoCA). Multiple logistic regression models were used to estimate the associations of serum sodium and frailty with MCI.

**Results:**

Participants with the lowest tertile of serum sodium had a higher risk of MCI than those in the middle tertile group (OR = 1.75, 95% CI: 1.01–3.04). Below 143 mmol/L, the risk of MCI was 1.38 (95% CI: 1.03–1.84) for per 1 SD decrease in serum sodium. Compared with the robust group, frailty was significantly associated with an increased risk of MCI (OR = 3.94, 95% CI: 1.92–8.10). Moreover, in comparison with participants with the middle tertile of serum sodium and who were robust/prefrail, those with frailty and either the lowest (OR = 5.53, 95% CI: 2.08–14.67) or the highest tertile of serum sodium (OR = 3.48, 95% CI: 1.20–10.05) had higher risks of MCI.

**Conclusion:**

Both lower and higher serum sodium impose a significantly higher risk for MCI in older adults with frailty. This could inform the design of clinical trials and the development of guidelines and recommendations for correcting serum sodium and frailty in hospitalized older adults with chronic diseases.

## 1 Introduction

Dementia is a progressive neurodegenerative syndrome characterized by acquired deterioration in cognition, function, and behavior, which interferes with independence in daily activities (1). It has been estimated that approximately 57.4 million people worldwide were affected by dementia in 2019 and this number is projected to reach 152.8 million by 2050 (2). With the unprecedented aging of the population, dementia has imposed a tremendous burden on health care resources and significantly impaired the quality of life among affected individuals and their families (3). Despite advances in symptomatic treatments, curative or effective disease-modifying therapies for dementia are lacking (4), thereby making prevention of this disease a major public health priority (5). As a preclinical, transitional state between healthy cognitive aging and dementia (6), mild cognitive impairment (MCI) represents a window of opportunity for intervention in which the impairments of one or more cognitive domains occur with specific neuropathological and biomarker changes (7). Therefore, a better understanding of the potentially modifiable risk factors for MCI is urgently needed to prevent or postpone the onset of dementia (8).

Hypoperfusion and hypometabolism in temporoparietal cortices have been recognized as pivotal neurobiological features of MCI (7). As the primary cation in extracellular fluid, sodium ion regulates the water-electrolyte balance, blood volume, and osmotic pressure (9). Abnormalities of serum sodium concentrations can trigger an imbalance of osmotic pressure between the outside and inside of the cell, potentially leading to disturbances in cerebral blood volume and neuronal activities (10, 11). Additionally, the underlying pathophysiological mechanisms of neurodegeneration involve oxidative stress and inflammation (12), both of which can be activated by altered sodium homeostasis (13, 14). Several epidemiological studies have revealed that lower serum sodium was associated with cognitive decline among older persons (15, 16); however, research conducted in the general population found null associations between lower circulating sodium levels and worse overall cognitive function (17).

The homeostasis of serum sodium can be profoundly disturbed by chronic diseases (18, 19), which also constitute significant risk factors for frailty, particularly in older adults (20). As a geriatric syndrome characterized by the accumulation of deficits (20), frailty also encompasses the domain of nutrition. It has been reported that older adults with nutritional frailty, the co-occurrence of physical frailty and nutritional imbalance which includes high dietary sodium intake (21), are at higher risk for all-cause mortality (22). Moreover, frailty shares common biological pathways with cognitive impairment, including elevations in biomarkers of oxidative stress and inflammatory cytokines (23). Numerous studies have shown a significant association between frailty and the risk of cognitive decline (24–26). Individuals with frailty experience more severe health consequences owing to declines in physiological reserves across multiple organ systems (20) and may exhibit impaired compensatory responses to sodium imbalances. Nevertheless, the joint associations of frailty and circulating sodium levels with MCI risks remain unexplored.

Therefore, this study aimed to investigate the associations of serum sodium concentrations as well as frailty with the risk of MCI, among Chinese hospitalized older adults with chronic diseases. Additionally, this study sought to determine whether the association between serum sodium levels and MCI risk varies according to frailty status. It was hypothesized that serum sodium concentrations and frailty are associated with MCI risk, and furthermore, the association between serum sodium and MCI risk depends on the frailty status of the individuals.

## 2 Materials and methods

### 2.1 Participants

**Figure 1:**
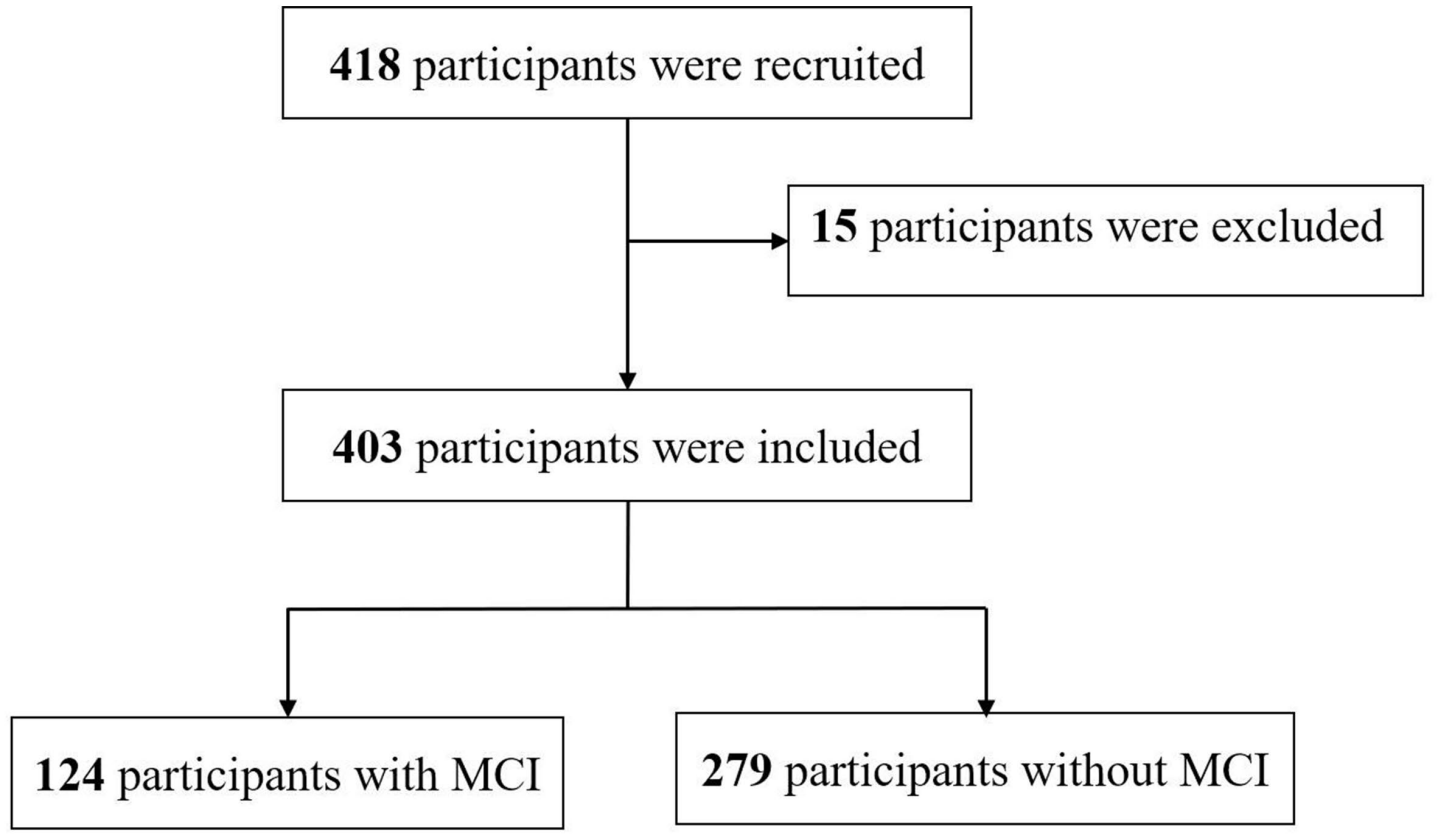
Flowchart of the study population.

A total of 418 participants were recruited and screened by research nurses in three tertiary hospitals in Xi’an, China. Structured questionnaires collected information on sociodemographic characteristics, anthropometric variables, lifestyle behaviors, disease status, and cognitive function at hospital admission. Peripheral fasting blood samples of the participants were extracted by trained registered nurses to determine serum sodium concentrations. Participants aged 60 years or older were eligible if they had been diagnosed with chronic diseases (e.g., coronary heart disease, hypertension, stroke, or type 2 diabetes) by a physician following the International Classification of Diseases, 10th revision (27). Individuals were excluded if they were diagnosed with any dementia (e.g., Alzheimer’s disease, vascular dementia, frontotemporal dementia, or Lewy body dementia), were suffering from other serious psychiatric conditions (e.g., schizophrenia, bipolar disorder, or intellectual disability), were currently taking psychotropic medications (e.g., benzodiazepines, anticholinergic medications, antidepressants), exhibited fluid and electrolyte imbalance upon hospital admission, had a previous history of head trauma, were unable to participate fully in the proposed assessment due to any consciousness or speech impairment, underwent surgery within the past 3 months, or had a life expectancy less than 6 months. After exclusion, 403 participants were included in the study analyses (Figure 1).

### 2.2 Assessment of serum sodium

Peripheral fasting blood samples for each participant were collected in vacutainers coated with Ethylene Diamine Tetraacetic Acid by phlebotomists during the first 24 h after hospital admission. Specimens were immediately centrifuged, aliquoted, and then stored in separator tubes at 4°C until assay. Serum concentrations of sodium were measured by ion selective electrode methodology using the Beckman AU5431 Chemistry Analyzer (Diamond Diagnostics, Holliston, MA, United States) by laboratory physicians following the standard operating procedures (28).

### 2.3 Assessment of frailty

Frailty was assessed by trained researchers using the FRAIL scale, which comprises five components: fatigue, resistance, ambulation, illnesses, and loss of weight (29). Participants met the criterion for fatigue if they responded “all of the time” or “most of the time” to the question, “How much time during the past 4 weeks have you felt tired?” Resistance was measured by asking participants if they had any difficulties in walking up 10 steps alone without resting and without aids, and ambulation was measured by asking if they had any difficulties in walking 100 meters alone and without aids. Participants met the criterion for illnesses if they reported five or more illnesses out of 11 total illnesses: hypertension, diabetes, cancer, chronic lung disease, heart attack, heart failure, angina, asthma, arthritis, stroke, and kidney disease. Loss of weight was measured by asking if participants had a weight reduction of 5% or more within the past 12 months. Participants who met 3 or more criteria were considered as frail, 1–2 criteria as pre-frail, and 0 as robust.

### 2.4 Assessment of MCI

MCI was determined by trained researchers using the Montreal Cognitive Assessment (MoCA), which is a brief cognitive screening tool that provides a global impression of a person’s cognitive integrity (30). It consists of 30 items and assesses short-term memory, visuospatial abilities, executive functions, attention, concentration and working memory, language, and orientation to time and place (31). The total MoCA scores were calculated by summing up the scores of each item and ranged from 0 to 30, with higher scores indicating a better cognitive function. The cut-off scores for MCI were 19 for individuals with no more than 6 years of education, 22 for individuals with 7 to 12 years of education, and 24 for individuals with more than 12 years of education (32).

### 2.5 Assessment of covariates

For each participant, the sociodemographic characteristics, anthropometric measurements, lifestyle behaviors, and disease status were selected *a priori* as potential covariates and were obtained by a self-administered questionnaire. Sociodemographic characteristics included age, gender, educational attainment, marital status, and socioeconomic status using *per capita* monthly household income. Anthropometric measurements included height and weight, which were measured by trained registered nurses following standard protocols at hospital admission. Body mass index (BMI) was calculated by dividing the participant’s weight in kilograms by their height in meters squared (kg/m^2^). Lifestyle behaviors included smoking and alcohol consumption. Chronic diseases (e.g., coronary heart disease, hypertension, stroke, and type 2 diabetes) were determined by self-report of diagnosis by a physician or other health care provider.

### 2.6 Sample size calculation and statistical analyses

The sample size was determined using the software package PASS version 14.0. As described previously (33), the prevalence of MCI was assumed to be 20.4% in Chinese older inpatients. With a relative precision of 20%, it was estimated that at least 374 participants should be recruited to achieve a power of 80% at a two-tailed significance level of 5%.

The normality of data distribution was tested using the Kolmogorov–Smirnov normality test. Normally distributed variables were described as mean and standard deviation (SD), while non-normally distributed variables were represented by the median and interquartile range (IQR). Categorical variables were expressed by frequency with percentage. According to the distribution of serum sodium, participants were categorized into tertiles, with the second tertile serving as the reference group. The Chi-square test and Fisher’s exact test were used for group comparisons.

To ascertain the association of serum sodium with MCI risks, odds ratios (ORs) with 95% confidence intervals (CIs) were estimated using logistic regression models. Three different models were performed: a crude model (Model 1), a gender- (men or women) and age-adjusted model (continuous) (Model 2), and a model in which we additionally adjusted for BMI (<18.5, 18.5–23.9, 24.0–27.9, or ≥ 28.0) (34), smoking status (never, past, or current), and alcohol consumption (never, past, or current) (Model 3). Dose–response relationships of serum sodium concentration with MCI risks were investigated graphically by restricted cubic splines with four knots. Similar logistical regression models were conducted to examine the association between frailty status and MCI risks. Tests for linear trend across the frailty status categories using the Wald test were performed by treating the three categories of frailty status as a continuous variable in the regression models. When exploring the joint association of frailty status and serum sodium, frailty status was merged into two categories: robust/prefrailty and frailty. To evaluate the robustness of the study findings, a sensitivity analysis was conducted by excluding the participants with cancer.

All statistical analyses were performed with the R packages (version 4.3.2). Two-tailed *p* < 0.05 was considered statistically significant.

## 3 Results

The characteristics of the study participants are presented in Table 1. Compared with participants in the middle tertile of serum sodium, those in the lowest and highest tertile groups were less likely to be married and to have a history of stroke. Lower serum sodium levels were positively associated with smoking and alcohol consumption. In addition, participants with frailty were older and tended to be non-drinkers, in contrast to those who were robust or prefrail.

**Table 1:** Characteristics of the participants by serum sodium and frailty status^a^.

Characteristics	Sodium levels (mmol/L)			*p*	Frailty status			*p*
	T1 (<141.2)	T2 (141.2–143.5)	T3 (>143.5)		Robust	Pre-frailty	Frailty	
No. of participants	138	135	130		154	196	53	
Mean (SD) age, years	71.6 (7.8)	69.9 (7.9)	71.7 (7.8)	0.09	69.8 (7.4)	70.4 (7.5)	77.2 (7.5)	**<0.01**
Male	88 (63.8)	66 (48.9)	42 (32.3)	**<0.01**	85 (55.2)	88 (44.9)	23 (43.4)	0.12
Married	95 (68.8)	113 (83.7)	96 (73.8)	**0.02**	119 (77.3)	152 (77.6)	33 (62.3)	0.06
Educational attainment				0.54				0.76
Less than college	101 (73.2)	91 (67.4)	89 (68.5)		105 (68.2)	137 (69.9)	39 (73.6)	
College or above	37 (26.8)	44 (32.6)	41 (31.5)		49 (31.8)	59 (30.1)	14 (26.4)	
BMI, kg/m^2^				0.54				0.78^b^
<18.5	7 (5.1)	8 (5.9)	10 (7.7)		8 (5.2)	13 (6.6)	4 (7.5)	
18.5–23.9	71 (51.4)	61 (45.2)	70 (53.8)		72 (46.8)	101 (51.5)	29 (54.7)	
24.0–27.9	51 (37.0)	55 (40.7)	38 (29.2)		61 (39.6)	68 (34.7)	15 (28.3)	
≥28	9 (6.5)	11 (8.1)	12 (9.2)		13 (8.4)	14 (7.1)	5 (9.4)	
Socioeconomic status^c^				0.24				0.20
Low	48 (34.8)	31 (23.0)	34 (26.2)		52 (33.8)	45 (23.0)	16 (30.2)	
Middle	47 (34.1)	57 (42.2)	55 (42.3)		55 (35.7)	81 (41.3)	23 (43.4)	
High	43 (31.2)	47 (34.8)	41 (31.5)		47 (30.5)	70 (35.7)	14 (26.4)	
Cigarette smoking				**<0.01**				0.29
Never	79 (57.2)	85 (63.0)	106 (81.5)		96 (62.3)	137 (69.9)	37 (69.8)	
Past/Current	59 (42.8)	50 (37.0)	24 (18.5)		58 (37.7)	59 (30.1)	16 (30.2)	
Alcohol consumption				**<0.01**				
Never	87 (63.0)	91 (67.4)	104 (80.0)		97 (63.0)	141 (71.9)	44 (83.0)	**0.02**
Past/Current	51 (37.0)	44 (32.6)	26 (20.0)		57 (37.0)	55 (28.1)	9 (17.0)	
T2D	29 (21.0)	30 (22.2)	34 (26.2)	0.58	32 (20.8)	44 (22.4)	17 (32.1)	0.23
Coronary heart disease	18 (13.0)	18 (13.3)	21 (16.2)	0.73	26 (16.9)	26 (13.3)	5 (9.4)	0.36
Stroke	12 (8.7)	18 (13.3)	6 (4.6)	**0.04**	13 (8.4)	18 (9.2)	5 (9.4)	0.96
Hypertension	18 (13.0)	21 (15.6)	25 (19.2)	0.38	25 (16.2)	31 (15.8)	8 (15.1)	0.98

T1 = tertile 1, T2 = tertile 2, T3 = tertile 3, BMI = body mass index, T2D = type 2 diabetes.

^a^Values are frequency (percentages) unless stated otherwise.

b Fisher’s exact test.

c Socioeconomic status represents per capita monthly household income.

Bold values indicate statistical significance.

**Figure 2:**
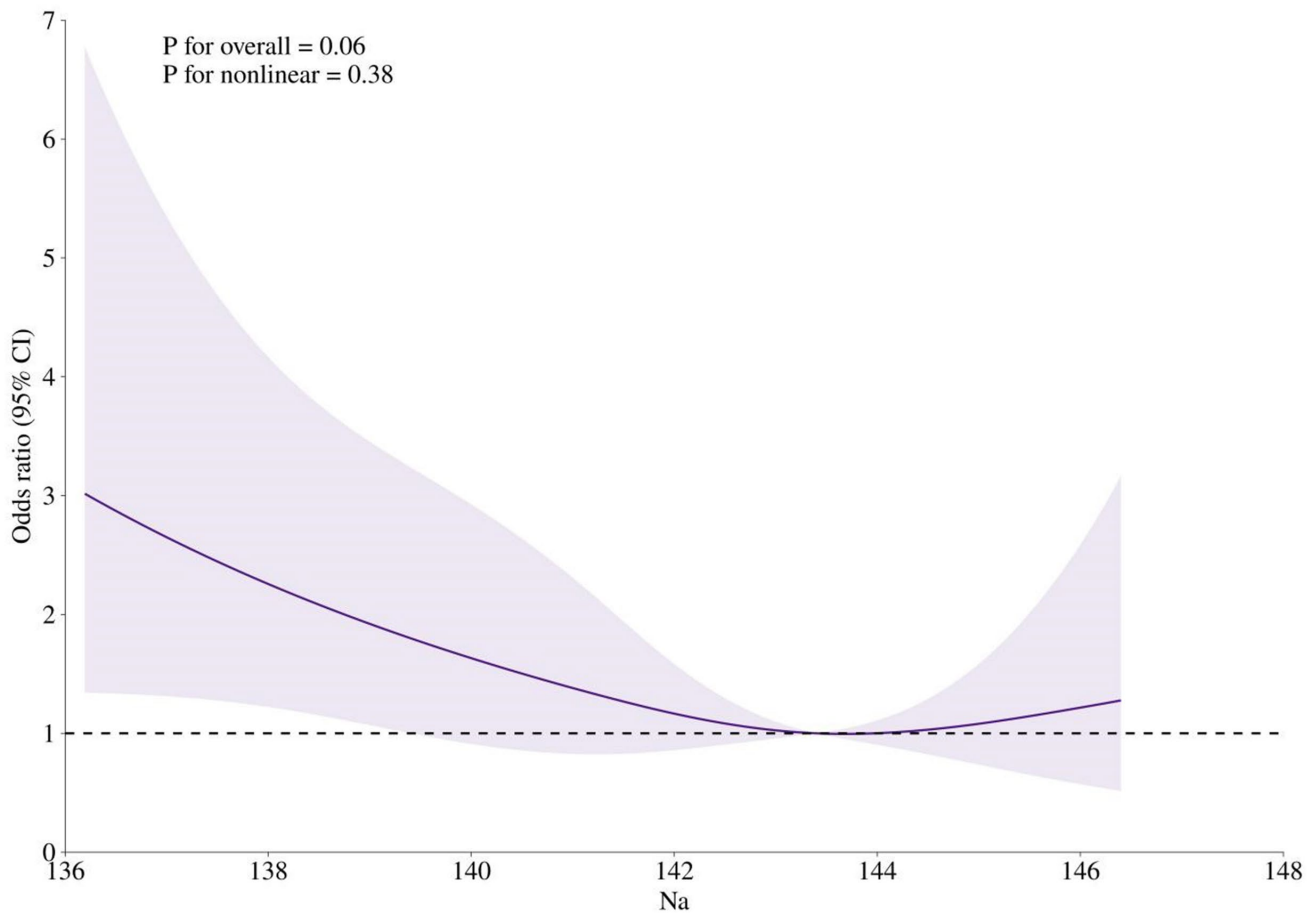
Restricted cubic spline analysis of the association between serum sodium concentration and MCI risk. Odds ratios were calculated in logistic models after adjusting for gender (men or women), age (continuous), BMI (<18.5, 18.5–23.9, 24.0–27.9, or ≥28.0), smoking status (never, past, or current), and alcohol consumption (never, past, or current).

Both the lowest and highest tertiles of serum sodium were positively associated with the risk of MCI, with OR of 1.91 and 1.14, respectively; however, only the association between the lowest tertile of serum sodium and MCI risk reached statistical significance (*p* = 0.01). Adjustments for gender and age somewhat attenuated these associations. After further controlling for BMI, smoking status, and alcohol consumption, participants in the lowest tertile of serum sodium had a 1.77-fold increased risk of MCI (95% CI: 1.02–3.06) compared with those in the middle tertile, whereas no statistically significant associations were observed between the highest tertile of serum sodium and MCI risk (OR = 0.99, 95% CI: 0.55–1.77) (Table 2). Restricted cubic spline analysis demonstrated a U-shaped relationship between serum sodium concentrations and MCI risk, with a nadir at approximately 143 mmol/L (Figure 2). Below 143 mmol/L, the risk of MCI was 1.38 (95% CI: 1.03–1.84) for per 1 SD decrease in serum sodium.

**Table 2:** Associations of serum sodium and frailty with MCI risk.

Exposures	Odds ratio (95% CI)			*Ptrend*
Serum sodium	**T1**	**T2**	**T3**	
Median (mmol/L)	139.8	142.5	144.6	
No. of cases/total participants	54/138	34/135	36/130	
Model 1	**1.91 (1.14, 3.20)**	1 [Reference]	1.14 (0.66, 1.97)	–
Model 2	**1.78 (1.04, 3.06)**	1 [Reference]	1.02 (0.58, 1.80)	–
Model 3	**1.77 (1.02, 3.06)**	1 [Reference]	0.99 (0.55, 1.77)	**–**
**Frailty status**	**Robust**	**Prefrailty**	**Frailty**	
FRAIL score	0	1–2	≥3	
No. of cases/total participants	39/154	51/196	34/53	
Model 1	1 [Reference]	1.04 (0.64, 1.68)	**5.28 (2.70, 10.30)**	**<0.01**
Model 2	1 [Reference]	1.02 (0.62, 1.68)	**4.08 (2.00, 8.30)**	**<0.01**
Model 3	1 [Reference]	0.99 (0.60, 1.64)	**3.99 (1.95, 8.17)**	**<0.01**

MCI, mild cognitive impairment; CI, confidence interval; T1, tertile 1; T2, tertile 2; T3, tertile 3.

Model 1: crude model.

Model 2: adjusted for gender (men or women) and age (continuous).

Model 3: further adjusted for BMI (<18.5, 18.5–23.9, 24.0–27.9, or ≥ 28.0), smoking status (never, past, or current), and alcohol consumption (never, past, or current).

Bold values indicate statistical significance.

Regarding frailty status, a significantly positive association was observed between frailty and the risk of MCI (OR = 5.28, 95% CI: 2.70–10.30); however, no significant associations were found for prefrailty. The gender- and age-adjusted model yielded a similar pattern of associations. When BMI, smoking status, and alcohol consumption were further adjusted for, participants with frailty still demonstrated an increased risk of MCI compared with those who were robust (OR = 3.99, 95% CI: 1.95–8.17). Additionally, a significantly increasing linear trend was observed between frailty status and MCI risks (*P* for trend<0.01) (Table 2).

**Figure 3:**
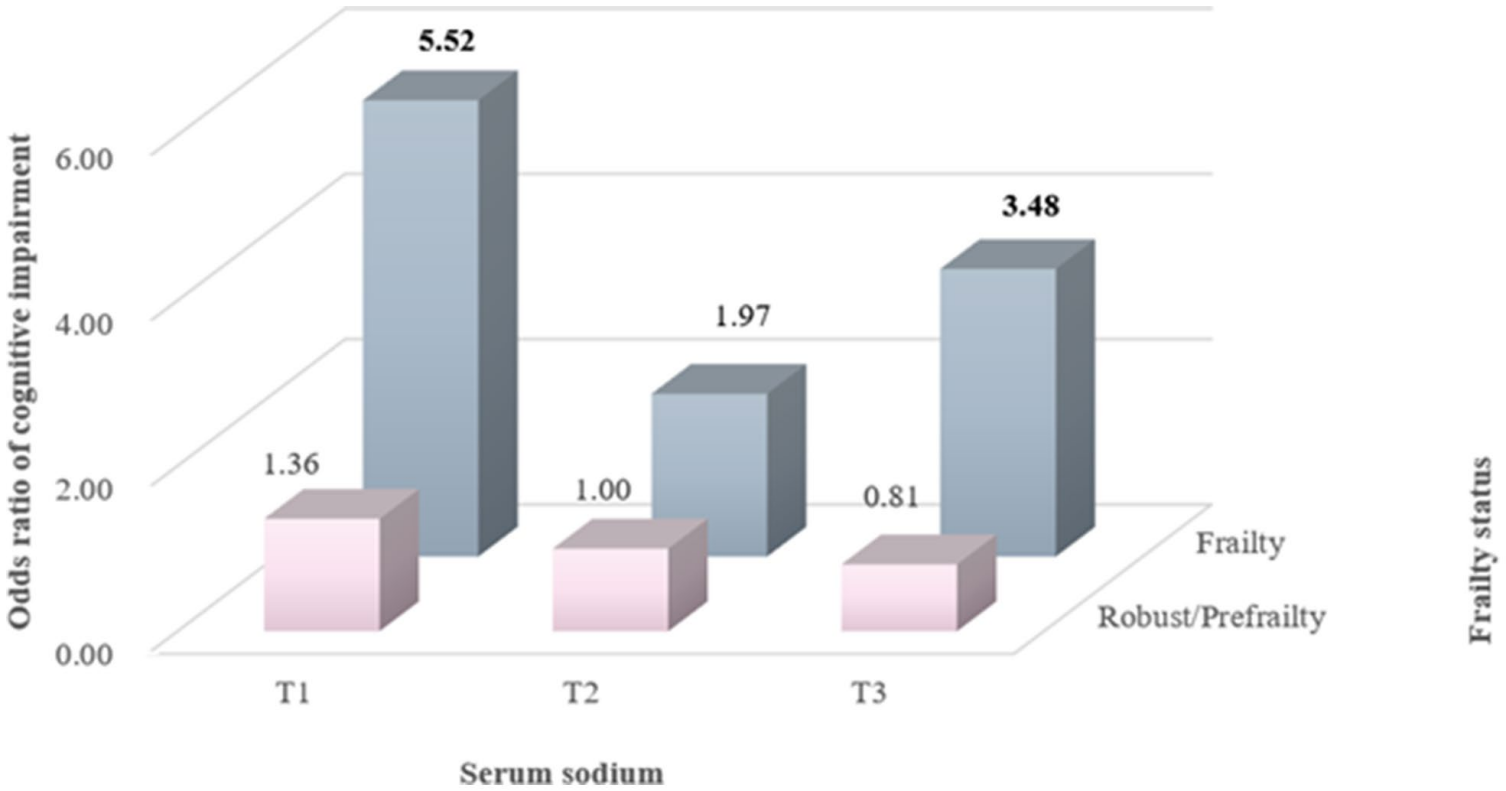
Joint association of serum sodium levels and frailty status with MCI risk. Odds ratios were calculated in logistic models after adjusting for gender (men or women), age (continuous), BMI (<18.5, 18.5–23.9, 24.0–27.9, or ≥28.0), smoking status (never, past, or current), and alcohol consumption (never, past, or current).

To estimate the joint association of serum sodium and frailty status with MCI risk, frailty status was dichotomized as robust/prefrailty and frailty, as prefrailty was not significantly associated with MCI risk. For participants who were robust or prefrail, both the lowest and highest tertile of serum sodium were not significantly associated with the risk of MCI. However, compared with the reference group of robust/prefrail participants in the middle tertile of serum sodium, those with frailty in the lowest and highest tertile of serum sodium demonstrated 5.52 (95% CI: 2.09–14.52) and 3.48 (95% CI: 1.20–10.07) times the risk of MCI, respectively (Figure 3).

In sensitivity analyses excluding 13 individuals with cancer, the associations of serum sodium and frailty status with MCI risk were largely similar to the results from primary analyses (Supplementary materials).

## 4 Discussion

The present study indicates that lower serum sodium concentration and frailty are associated with an elevated risk of MCI. A significantly positive association was also observed between higher serum sodium levels and the risk of MCI among individuals with frailty. These findings extended the existing evidence by demonstrating that the relationship between serum sodium and the risk of MCI varies according to frailty status.

Previous studies have examined the association between lower serum sodium and risks of cognitive deterioration. A cross-sectional study conducted in the elderly US population revealed that hyponatremia was significantly associated with cognitive change, particularly in memory and executive function (15). In a cohort study of older community-dwelling men, individuals with lower serum sodium levels had 1.30 times the odds of cognitive impairment compared to those with sodium levels of 141–142 mmol/L (16). Despite the heterogeneity in study samples and the various assessment instruments for cognitive function, the consistent findings across studies are that a reduction in serum sodium concentration can adversely impact cognitive functioning. Our study similarly suggests that lower serum sodium is significantly associated with an elevated risk of MCI among hospitalized older adults with chronic conditions. The neurological detriment of lower serum sodium can primarily be attributed to the abnormality of osmotic pressure in the brain. In response to hypotonicity, astrocyte swelling can induce the release of electrolytes and organic osmolytes (35), some of which act as excitatory neurotransmitters (e.g., glutamate) and play a crucial role in neuronal damage (10). Another potential mechanism linking lower serum sodium to MCI involves impaired neuronal mitochondrial distribution and decreased ATP production (36). In primary neurons cultured in a hyponatremic medium, Fujisawa et al. found that mitochondria disappeared from neurites, suggesting that chronic hyponatremia impairs mitochondrial function (36), which may deleteriously affect neuritic transport by regulating cellular energy production and oxidative stress control (37). In older adults, chronic hyponatremia is common but often rather mild and asymptomatic (38). Our results indicate that reduced serum sodium could constitute a modifiable target for preventing cognitive dysfunction, especially in older adults with chronic diseases.

A significant association between frailty and MCI risk was observed, aligning with previous evidence (23, 39, 40). For example, a cross-sectional study of Chinese middle-aged and elderly people found that frailty was significantly associated with lower Mini-mental State Examination scores and increased MCI risk (39). Similarly, cohort studies of community-dwelling older adults have demonstrated that frailty is associated with a subsequent decline in cognitive function within 12 months (23) or over 2 years (40). The possible mechanisms underlying the association of frailty and cognitive impairment may be diverse, including inflammation and oxidative stress. Chronic proinflammatory activation (e.g., IL-6, TNF-*α*) is a cardinal pathophysiologic process contributing to frailty (41), and inflammation has been implicated in the neuropathological cascade through modulating the amyloid protein precursor processing and increasing the production of amyloid-β42 peptide (42). Moreover, frailty is associated with oxidative imbalance due to overproduction or insufficient elimination of reactive oxygen species (43, 44). Compared to robust older adults, individuals with frailty have higher levels of oxidative stress (45), which may trigger synaptic dysfunction and cognitive decline (46, 47). It has been proposed that the detrimental association between frailty and cognitive impairment could be bidirectional (48). Therefore, interventions aimed at improving components of frailty may mitigate the risk of MCI in hospitalized older adults with chronic diseases.

It should be noted that higher serum sodium was significantly associated with an elevated risk of MCI in older adults with frailty, while no associations were found for individuals who were robust or prefrail. Similarly, Wei and colleagues found that functional disability attributed to malnutrition were more likely to be associated with frailty (49). One potential mechanism of action for these divergent associations is that frailty could aggravate hyperosmotic stress and cell dehydration, severely impacting the structure and function of intracellular proteins (50). Our study provides complementary evidence supporting the notion that frailty represents a state of increased vulnerability to poor resolution of homeostasis following stress (20). Sodium is one of the most important extracellular ions for regulating osmotic pressure, which governs the exchange of water between intracellular and extracellular spaces (51). Once the body systems lose their in-built reserves, frailty could make cells more sensitive to elevated serum sodium, potentially contributing to dehydration (52). Suboptimal hydration may subsequently increase cortisol levels (53), which can affect memory function and speed of information processing (54). Another potential mechanism is pertinent to sodium-induced inflammation (55). Higher serum sodium may upregulate systemic inflammatory responses (14) and amplify the already elevated inflammatory responses in older adults with frailty (56), thereby synergistically exacerbating the neurotoxic effects of inflammation. The results of our study indicate that higher serum sodium can be a surrogate marker for MCI in older adults with frailty. Nonetheless, further studies are needed to elucidate the mechanism underlying the observed associations of serum sodium with MCI risk, particularly as modified by frailty status.

This study has several limitations that should be acknowledged when interpreting the findings. Firstly, the cross-sectional design precludes the establishment of causal relationships between exposures and health outcomes. Future research should employ longitudinal designs or randomized controlled trials to determine the temporal sequence of serum sodium levels, frailty, and MCI. Secondly, although several potential confounders were collected and controlled for in the multivariate models, we could not rule out the possibility that unmeasured confounders may have influenced the results. Thirdly, almost all the participants had a serum sodium level not exceeding the criteria of hyponatremia and hypernatremia, which may underestimate the detrimental effects of altered sodium homeostasis on cognitive functioning. Fourthly, because natremia is a highly variable value, and cerebral cells can respond to changes in serum osmolality to maintain homeostasis, a single, punctual value may not be sufficiently representative. Therefore, serum sodium variance over time should be considered when examining the effects of serum sodium on MCI risks. Fifthly, the MCI of participants was measured by the MoCA scale only, a brief screening tool for global cognitive function, rather than a combination with comprehensive neuropsychological test assessments. However, this tool has indicated high sensitivity and specificity for the detection of MCI in various populations (57, 58). Finally, the findings of this study, conducted among hospitalized older adults with chronic diseases, may not be generalizable to other populations due to the specific characteristics and conditions of the study sample.

## 5 Conclusion

In conclusion, participants with lower serum sodium or frailty are more likely to have MCI. If meeting the criteria of frailty, higher serum sodium also imposes an increased risk of MCI to hospitalized older adults with chronic diseases. Additional studies should be conducted to confirm whether the correction of lower and higher serum sodium in older adults with frailty improves their cognitive function. In addition, future studies are needed to examine if these associations are similar in other aging populations and to elucidate the mechanism underlying such relationships.

## Data Availability

The raw data supporting the conclusions of this article will be made available by the authors, without undue reservation.
