# Supplementary material for: Joint association of serum sodium and frailty with mild cognitive impairment among hospitalized older adults with chronic diseases: a cross-sectional study
Source: Front Nutr. 2024 Oct 21;11:1467751. doi: 10.3389/fnut.2024.1467751 (PMC11532049; doi:10.3389/fnut.2024.1467751)
Supplement: Supplementary file 1 [file Data_Sheet_1.docx]

Supplementary Material

**Table 1** Associations of serum sodium and frailty with MCI risk

| **Exposures** | **Odds ratio (95% CI)** | | | ***P_trend_*** |
| --- | --- | --- | --- | --- |
| **Serum sodium** | **T1** | **T2** | **T3** |  |
| Median (mmol/L) | 139.8 | 142.5 | 144.7 |  |
| No. of cases/total participants | 53/132 | 32/130 | 35/128 |  |
| Model 1 | **2.01 (1.21, 3.49)** | 1 [Reference] | 1.15 (0.66, 2.01) | – |
| Model 2 | **1.83 (1.05, 3.21)** | 1 [Reference] | 0.98 (0.54, 1.77) | – |
| Model 3 | **1.80 (1.02, 3.18)** | 1 [Reference] | 0.97 (0.53, 1.77) | **–** |
| **Frailty status** | **Robust** | **Prefrailty** | **Frailty** |  |
| FRAIL score | 0 | 1–2 | ≥3 |  |
| No. of cases/total participants | 38/152 | 49/187 | 33/51 |  |
| Model 1 | 1 [Reference] | 1.07 (0.65, 1.68) | **5.50 (2.78, 10.87)** | **<0.01** |
| Model 2 | 1 [Reference] | 1.07 (0.64, 1.78) | **3.73 (1.81, 7.70)** | **<0.01** |
| Model 3 | 1 [Reference] | 1.05 (0.63, 1.76) | **3.62 (1.73, 7.56)** | **<0.01** |

*Notes*: MCI=mild cognitive impairment, CI=confidence interval, T1=tertile 1, T2=tertile 2, T3=tertile 3.

Model 1: crude model.

Model 2: adjusted for gender (men or women) and age (continuous).

Model 3: further adjusted for BMI (<18.5, 18.5–23.9, 24.0–27.9, or ≥28.0), smoking status (never, past, or current), and alcohol consumption (never, past, or current).


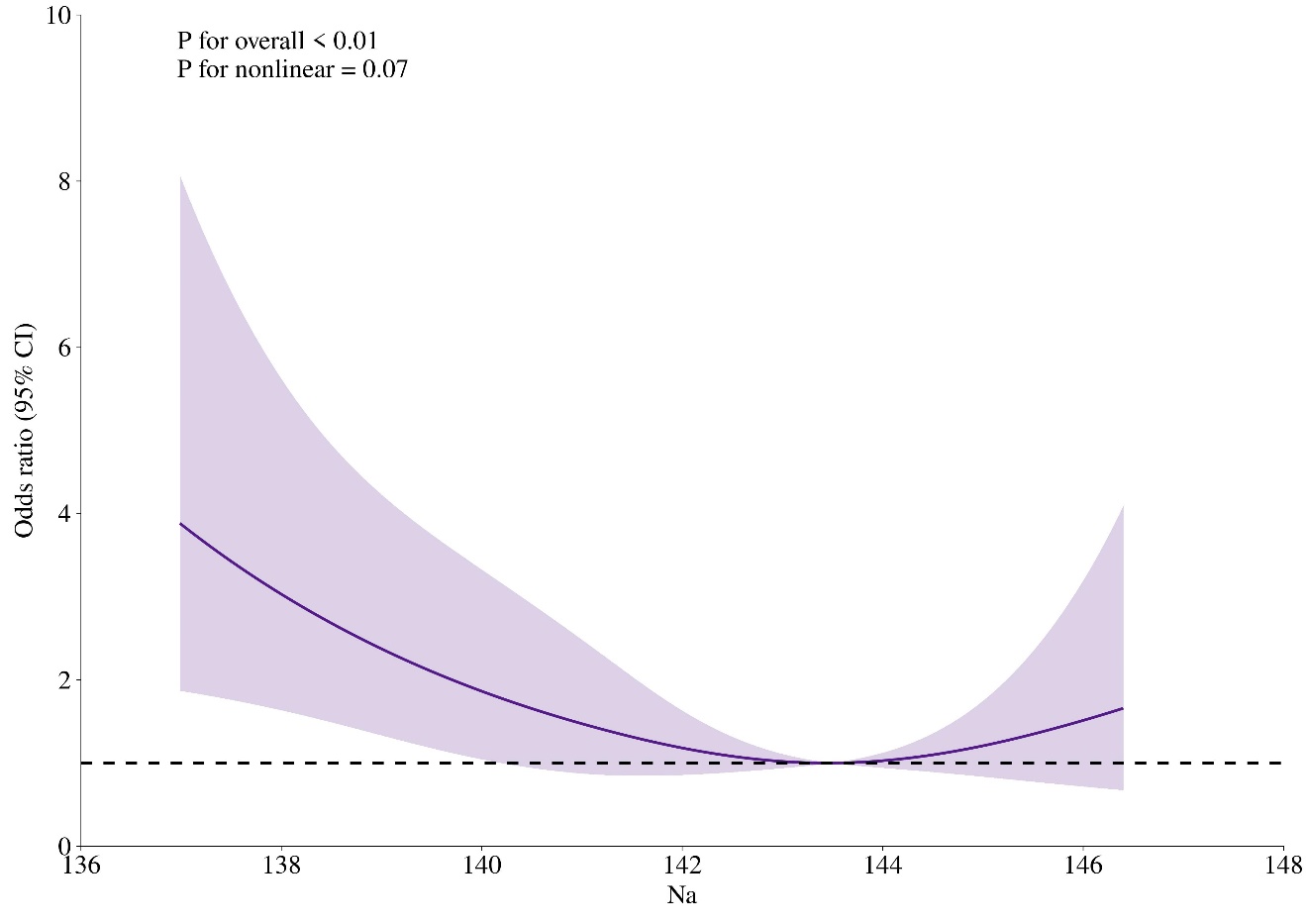
**Figure 1** Restricted cubic spline analysis of the association between serum sodium concentration and MCI risk. *Notes:* Odds ratios were calculated in logistic models after adjusting for gender (men or women), age (continuous), BMI (<18.5, 18.5–23.9, 24.0–27.9, or ≥28.0), smoking status (never, past, or current), and alcohol consumption (never, past, or current)


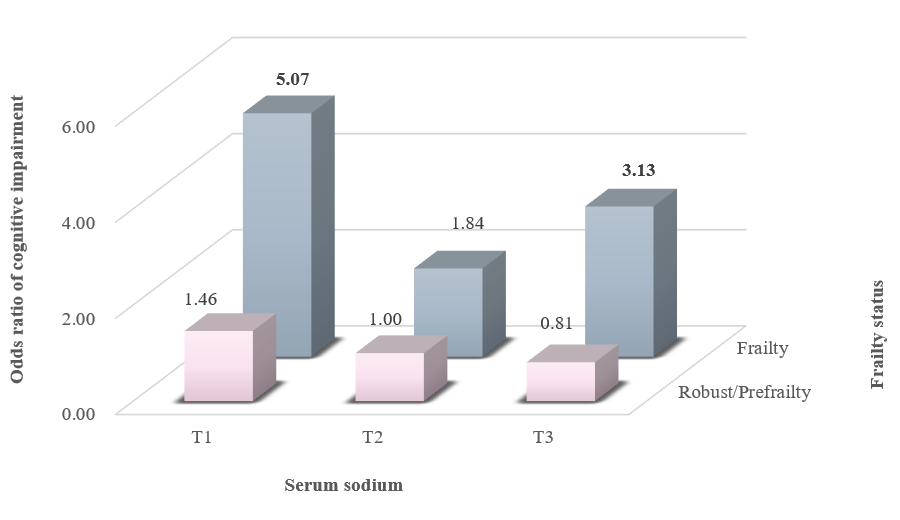


**Figure 2** Joint association of serum sodium levels and frailty status with MCI risk. *Notes*: Odds ratios were calculated in logistic models after adjusting for gender (men or women), age (continuous), BMI (<18.5, 18.5–23.9, 24.0–27.9, or ≥28.0), smoking status (never, past, or current), and alcohol consumption (never, past, or current).
